# Immune Monitoring of Patients With Primary Immune Regulation Disorders Unravels Higher Frequencies of Follicular T Cells With Different Profiles That Associate With Alterations in B Cell Subsets

**DOI:** 10.3389/fimmu.2020.576724

**Published:** 2020-10-29

**Authors:** María Soledad Caldirola, María Paula Martínez, Liliana Bezrodnik, Norberto Walter Zwirner, María Isabel Gaillard

**Affiliations:** ^1^Inmunología, Instituto Multidisciplinario de Investigaciones en Patologías Pediátricas (IMIPP- CONICET-GCBA)–Hospital de Niños “Ricardo Gutiérrez”, Buenos Aires, Argentina; ^2^Centro de Inmunología Clínica Dra. Bezrodnik, Buenos Aires, Argentina; ^3^Instituto de Biología y Medicina Experimental (IBYME-CONICET), Laboratorio de Fisiopatología de la Inmunidad Innata, Buenos Aires, Argentina; ^4^Departamento de Química Biológica, Facultad de Ciencias Exactas y Naturales, Universidad de Buenos Aires, Buenos Aires, Argentina; ^5^Sección Citometría–Laboratorio Stamboulian, Buenos Aires, Argentina

**Keywords:** primary immunodeficiencies, follicular helper T cells, flow cytometry, switched memory B cells, primary immune regulation disorders

## Abstract

Primary immune regulation disorders lead to autoimmunity, allergy and inflammatory conditions due to defects in the immune homeostasis affecting different T, B and NK cell subsets. To improve our understanding of these conditions, in this work we analyzed the T and B cell compartments of 15 PID patients with dysregulation, including 3 patients with STAT1 GOF mutation, 7 patients with CVID with dysregulation, 3 patients with mutations in CTLA4, 1 patient with CD25 mutation and 1 patient with STAT5b mutation and compared them with healthy donors and with CVID patients without dysregulation. CD4^+^ and CD8^+^ T cells from the patients exhibited a significant decreased frequency of naïve and regulatory T cells with increased frequencies of activated cells, central memory CD4^+^ T cells, effector memory CD8^+^ T cells and terminal effector CD8^+^ T cells. Patients also exhibited a significantly increased frequency of circulating CD4^+^ follicular helper T cells, with altered frequencies of cTfh cell subsets. Such cTfh cells were skewed toward cTfh1 cells in STAT1 GOF, CTLA4, and CVID patients, while the STAT5b deficient patient presented a skew toward cTfh17 cells. These alterations confirmed the existence of an imbalance in the cTfh1/cTfh17 ratio in these diseases. In addition, we unraveled a marked dysregulation in the B cell compartment, characterized by a prevalence of transitional and naïve B cells in STAT1 GOF and CVID patients, and of switched-memory B cells and plasmablast cells in the STAT5b deficient patient. Moreover, we observed a significant positive correlation between the frequencies cTfh17 cells and switched-memory B cells and between the frequency of switched-memory B cells and the serum IgG. Therefore, primary immunodeficiencies with dysregulation are characterized by a skew toward an activated/memory phenotype within the CD4^+^ and CD8^+^ T cell compartment, accompanied by abnormal frequencies of Tregs, cTfh, and their cTfh1 and cTfh17 subsets that likely impact on B cell help for antibody production, which likely contributes to their autoimmune and inflammatory conditions. Therefore, assessment of these alterations by flow cytometry constitutes a simple and straightforward manner to improve diagnosis of these complex clinical entities that may impact early diagnosis and patients’ treatment. Also, our findings unravel phenotypic alterations that might be associated, at least in part, with some of the clinical manifestations observed in these patients.

## Introduction

Primary Immune Regulation Disorders (PIRD)s lead to defects in the immune homeostasis that cause a defective or exacerbated immune response that usually produce autoimmunity, allergy, and/or inflammation (1–6). These diseases constitute an expanding group of primary immunodeficiencies (PID) listed in the last IUIS Phenotypic Classification of PID (7). Within this group, those that course with autoimmunity are the result of defects in regulatory T cell development and/or function (8–10), whose hallmark disease is immune dysregulation, polyendocrinopathy, enteropathy, X-linked (IPEX), a disease caused by mutations in the *FOXP3* gene (11–13). However, two thirds of patients with a phenotype that resembles IPEX do not exhibit FOXP3 mutations. Deleterious mutations in IL-2RA (CD25), STAT5b, CTLA-4, LRBA (14–17), and gain of function mutations in STAT1 and STAT3 (18–20) have been described as causing IPEX-like syndromes. Many patients with common variable immune deficiency (CVID) may also present an IPEX-like phenotype, as they can present cytopenias, inflammatory bowel disease, allergies, granulomas, lymphoproliferation, and/or malignancies (21). Autoimmunity can be the first sign of immune dysregulation, even preceding other manifestations such as susceptibility to specific infectious organisms (5, 22).

Follicular helper T cells (Tfh) cells were originally described in human tonsils as a separate subset of memory CD4^+^ T cells expressing the chemokine receptor CXCR5, specialized in providing help to B cells (23, 24). They are essential for the formation of germinal centers (GC), where B cells become activated and differentiate into long-lived memory B lymphocytes (MBL) and plasmablast cells (PBC) (25–28). Some studies reported that a small counterpart of Tfh circulate in peripheral blood, and they were named “circulating Tfh cells” (cTfh) (29–31). Analyses of cTfh revealed that they contain different subsets with unique phenotypical and functional characteristics (32, 33). According to CXCR3 and CCR6 expression, transcription factors and cytokines produced, cTfh cells were classified into cTfh1 (CXCR3^+^CCR6^-^), cTfh2 (CXCR3^-^CCR6^-^) and cTfh17 (CXCR3^-^CCR6^+^) cells, resembling the classical Th1, Th2, and Th17 cell subsets (26, 32). Only cTfh17 and cTfh2 cells are highly efficient for B cell help due to their production of IL-21 (34, 35). Also, Tfh cells play a crucial role in the long-term maintenance of antibody production that, in the case of antibody-mediated autoimmune diseases, may contribute to the pathogenesis of these diseases (25, 35, 36). Moreover, phenotypical abnormalities in different T cell, B cell, and NK cell compartments might be associated with several clinical findings usually observed in PID with immune dysregulation (37–41), and their characterization may contribute to a better identification or classification of PID patients (42–44). Such alterations might be assessed by flow cytometry (FC), especially in some institutions were next generation sequencing (NGS) is not available. Furthermore, FC is in fact a quicker, useful and less expensive tool that may guide clinician’s diagnostic suspicion (31, 42, 44).

Therefore, in this work, we performed a characterization of T and B cell subsets of a cohort of 15 patients with PIRD and analyzed whether such alterations are associated their clinical features.

## Materials and Methods

### Samples

Samples from 15 patients with PID with immune dysregulation were included: 1 patient with CD25 deficiency (Y41S mutation) (14), 1 patient with STAT5b mutation (F646S) (15), 3 patients with STAT1 gain of function (GOF) mutations (Q167H, R274Q, and F172L), 3 family-related patients with the same CTLA4 mutation (L141P) and 7 CVID (ESID criteria) patients with dysregulation with unknown molecular defect (CVID_dys_). The cohort included 9 female and 6 male patients with a median age of 25.7 years (range: 12–48 years). The main clinical features of these patients are summarized in **Supplementary Table 1**. Assessment of T and B cell subsets was repeated at least twice and evaluated prior treatment and after one year following cessation of immunosuppression when possible; immunosuppressive and/or immunomodulator therapies are detailed in **Supplementary Table 1**. As controls, we included a group of healthy donors (HD) and a group of five CVID patients without immune dysregulation (CVID_no-dys_). Whole blood was collected by venipuncture in tubes with EDTA. Samples from healthy volunteers were provided by the Blood Bank of the “Ricardo Gutiérrez” Children’s Hospital (Buenos Aires, Argentina). Studies have been approved by the institutional review committee and informed consent of participating subjects or their legal guardians if they were minors was obtained.

### Antibodies and Reagents

The following fluorochrome-labeled monoclonal antibodies (mAb) against human molecules were used: APC-anti-CD3 (SK7), PerCP/Cy5.5-anti-CD4 (SK3), APC-H7-anti-CD8 (SK1), PE-anti-HLA-DR (TU36), PE-Cy7-anti-CD19 (SJ25C1), FITC-anti-CD45RA (L48), Brilliant Violet 421-anti-CD27 (M-T271), PE-anti-CD25 (2A3), Alexa 488-anti-FOXP3 (259D/C7), Pacific Blue-anti-CD4 (RPA-T4), PE-Cy5-anti-CD21 (B-ly4), FITC-anti-IgD (IA6-2), APC-anti-IgM (G20-127), APC-H7-anti-CD38 (HB7), PE-anti-CD24 (ML5) from BD; APC-anti-CXCR5 (J252D4), PE-Cy7-anti-CD45RA (HI100), Pacific Blue-anti-CXCR3 (G025H7), and Brilliant Violet-anti-CCR6 (G034E3) from BioLegend.

### Flow Cytometry

Immunostaining of T cells was performed using 100 μl of whole blood collected with EDTA and stained during 15 min at room temperature with the corresponding mAb. Thereafter, red blood cells were lysed using FACS Lysing Solution (BD) for 7 min, washed twice with PBS/BSA and acquired. After gating on CD45^+^ cells, followed by a second gate on CD3^+^ cells, subsets within the CD4^+^ and CD8^+^ cells were defined as naïve T cells (CD45RA^+^CD27^+^), central memory T cells (T_CM_, CD45RA^-^CD27^+^), effector memory T cells (T_EM_, CD45RA^-^CD27^-^) and terminal effector T cells (T_EMRA_, CD45RA^+^CD27^-^), respectively as described (45–47). Activated CD4^+^ and CD8^+^ T cells were characterized as HLA-DR^+^ cells. cTfh (CD4^+^CD45RA^-^CXCR5^+^) cells were divided in 2 subsets: cTfh1 (CCR6^-^CXCR3^+^) and cTfh17 (CCR6^+^CXCR3^-^) (30, 34). For B lymphocytes (BL), 250 μl of whole blood collected with EDTA were lysed using Pharm Lyse (BD), washed twice with PBS/BSA and stained with the corresponding mAb for 30 min at room temperature. Thereafter, a second lysis with FACS Lysing Solution (BD) for 7 min was performed, cells were washed twice with PBS/BSA and acquired. Different stages of B cell differentiation were identified within the CD19^+^ cell subpopulation as transitional or immature B cells (TBL, CD38^++^CD24^++^), naïve B cells (IgD^+^CD27^-^), CD21^low^ B cells (CD21^low^CD38^+/-^), post-switched MBL (Sw-MBL, IgD^-^IgM^-^CD38^+/-^), and plasmablasts (PBC, CD38^++^CD27^++^), as described (48). For regulatory T cells (Treg, CD4^+^CD25^++^FOXP3^+^), 1 × 10^6^ PBMC/mm^3^ were labeled with the corresponding mAb following the manufacturer protocol (Anti-Human FOXP3 Staining Kit, BD). Cells were acquired in a FACSCanto II flow cytometer (BD) and analyzed using the FlowJo software v10.0.7 (Treestar, Inc.).

### Statistical Analysis

All data are presented as relative values. The differences between HD and patients as a whole group were analyzed by an unpaired *t*-Student tests. If the group passed the normality test, a parametric method (Welch test) was used. If the group did not pass the normality test, a non-parametric method (Mann-Whitney U test) was used. When more than two groups were compared and because in all cases at least one group did not pass normality test, a non-parametric Kruskal-Wallis test with Dunn’s *post hoc* test was used. Spearman correlation was used to assessed association between variants. A two-sided p-value of <0.05 was considered statistically significant. GraphPad Prism 6.01 (GraphPad Software) was used for all graphs and statistical analyses.

## Results

### CD4^+^ T Cells From PIRD Patients Exhibit Increased Frequencies of Activated, TCM and cTfh cells, Reduced Frequencies of Treg Cells, and Altered Frequencies of cTfh Cell Subsets

**Figure 1:**
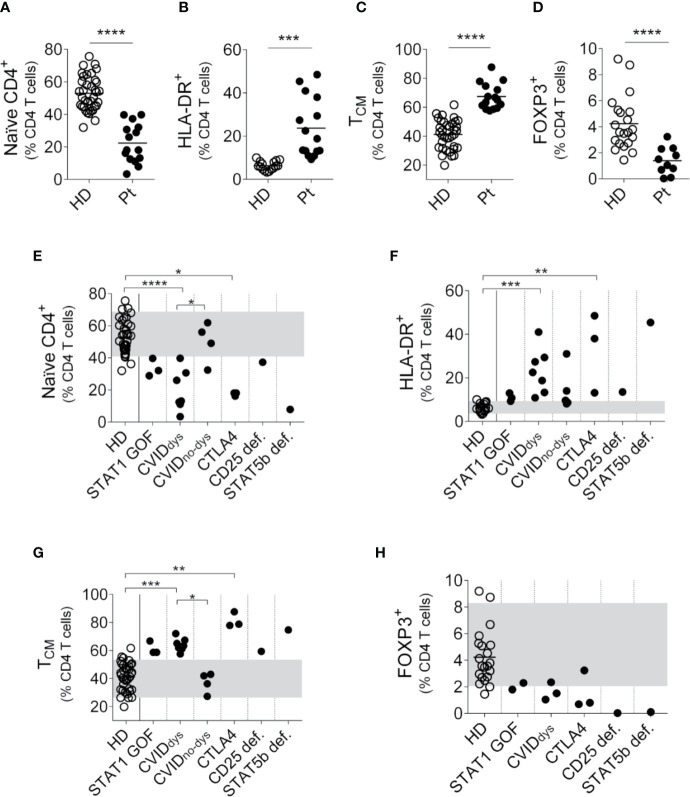
Relative frequencies of CD4^+^ T cells subsets in PIRD patients. The frequency of CD45RA^+^CD27^+^CD4^+^ (naïve) T cells **(A)**, HLA-DR^+^CD4^+^ (activated) T cells **(B)**, CD45RA^-^CD27^+^CD4^+^ (T_CM_, **C)** and regulatory T cells (Treg, CD4^+^CD25^++^FOXP3^+^, **D**) cells in a cohort of 36 HD **(A-C)**, 21 HD **(D)**, 15 patients with PIRD **(A–C)** and 10 patients with PIRD **(D)** were depicted. Horizontal lines represent the mean of each group. The distribution of the frequencies of CD45RA^+^CD27^+^CD4^+^ (naïve) T cells **(D)**, HLA-DR^+^CD4^+^ (activated) T cells **(E)**, CD45RA^-^CD27^+^CD4^+^ (T_CM_ cells, **F)** and CD4^+^CD25^++^FOXP3^+^ (Treg, **H)** in each group of this cohort was also depicted. P1 (STAT1 GOF) and P11 (CVID) were under treatment (as detailed in **Supplementary Table 1**). Gray areas indicate the 10^th^ and 90^th^ percentiles of HD for each parameter. An unpaired parametric *t*-Student test with Welch´s correction was used in **(A–D)**; a non-parametric test with Dunn’s *post hoc* was used in **(D–H)**. *p < 0.05; **p < 0.01; ***p < 0.001; ****p < 0.0001.

We first performed the analysis of naϊve and memory T cell subsets in a cohort of PIRD patients (**Figure 1**). To identify the different CD4^+^ T cell subpopulations, we use the gating strategy outlined in **Supplementary Figure 1A**. Analyzed as a group and compared to HD, CD4^+^ T cells from the patients exhibited a significant decreased frequency of naïve CD4^+^ T cells (**Figure 1A**) with increased frequencies of activated (HLA-DR^+^) CD4^+^ T cells (**Figure 1B**) and T_CM_ cells (**Figure 1C**). In addition, we did not observe significant differences in the frequency of T_EM_ (CD45RA^-^CD27^-^) and T_EMRA_ (CD45RA^+^CD27^-^) cells between patients and HD (*not shown*). Moreover, analysis of CD4^+^CD25^++^FOXP3^+^ cells using the gating strategy outlined in **Supplementary Figure 1B** confirmed that patients with dysregulation evidenced lower frequencies of Treg cells (**Figure 1D**). Disaggregated analysis of individual patients according to their mutation confirmed these differences, although they did not reach statistical significance in the STAT1 GOF patients (**Figures 1E–H**). Also, compared to the CVID_no-dys_ group, CVID_dys_ patients presented lower frequencies of naïve CD4^+^ T cells (**Figure 1E**) and higher frequencies of T_CM_ cells (Figure G). Of note, although P2 and P3 (STAT1 GOF), P4 (STAT5b def.), P5 (CD25 def), P6 (CTLA4), P9, and P10 (CVID) exhibited intermittent total and/or CD4^+^ T lymphopenia *(not shown)*.

**Figure 2:**
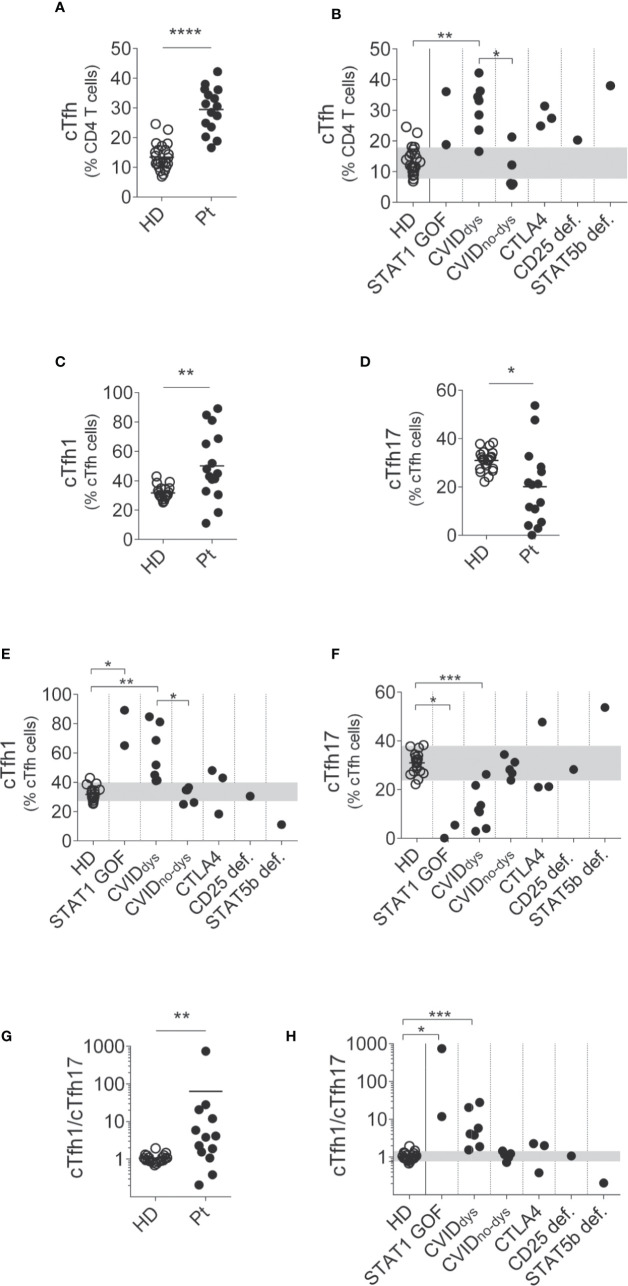
Relative frequencies of cTfh and their subsets in PIRD patients. The frequency of CD4^+^CD45RA^-^CXCR5^+^ cTfh cells in a cohort of 24 HD and 14 patients with dysregulatory syndrome PID **(A)** and their distribution in each group of this cohort **(B)** were depicted. Also, the frequencies of CCR6^-^CXCR3^+^ (cTfh1, **C**) and CCR6^+^CXCR3^-^ (cTfh17, **D)** cells within cTfh cells in this cohort, and the distribution of the frequencies of CCR6^-^CXCR3^+^ (cTfh1, **E**) and CCR6^+^CXCR3^-^ (cTfh17, **F**) cells in each group of this cohort were depicted. In addition, the cTfh1/cTfh17 ratio **(G)** and its distribution in each group of this cohort **(H)** are shown. Horizontal lines in **(A, C, D)** represent the mean of each group. P1 (STAT1 GOF) and P11 (CVID) were under treatment (as detailed in **Supplementary Table 1**). Gray areas in **(B, E, F)** indicate the 10^th^ and 90^th^ percentiles of HD for each parameter. An unpaired parametric *t*-Student test with Welch´s correction was used in **(A, C, D)**; a non-parametric test with Dunn’s *post hoc* was used in **(B, E, F)**. An unpaired non-parametric *t*-Student test with Mann-Whitney U test was used in **G**. *p < 0.05; **p < 0.01; ***p < 0.001; ****p < 0.0001.

Next, we evaluated the frequency of cTfh in peripheral blood (**Figure 2**). Although we could not perform this analysis on P3 because we lost follow up, in the rest of the patients we observed a significant increased frequency of cTfh compared to HD (**Figure 2A**). In addition, this difference was significant in the CVID_dys_ group when compared to HD and to the CVID_no-dys_ group (**Figure 2B**). An analysis of cTfh subpopulations revealed that the patients exhibited higher frequencies of cTfh1 cells (**Figure 2C**) and cTfh17 cells (**Figure 2D**). The disaggregated analysis revealed that, compared to HD, STAT1 GOF, and CVID_dys_ patients exhibited increased frequencies of cTfh1 cells, while the STAT5b deficient patient evidenced a reduced frequency of cTfh1 cells (**Figure 2E**). Remarkably, we also observed that CVID_dys_ patients exhibited an increased frequency of cTfh1 cells compared to CVID_no-dys_ patients. Moreover, compared to HD, STAT1 GOF, CVID, and 2 CTLA4 patients exhibited concomitant decreased frequencies of cTfh17 cells that were significant in the STAT1 GOF and CVID_dys_ patients (**Figure 2F**). However, although CVID_no-_dys patients exhibited frequencies of cTfh17 cells that were within the range of HD, differences in the frequencies of cTfh17 cells between both groups of CVID patients were not significant, likely due to the low number of patients that we recruited in this study. In addition, cTfh17 cells were almost absent in the STAT1 GOF and in two of the CVID patients, while the STAT5b deficient patient exhibited an increased frequency. Accordingly, patients with dysregulation exhibited a significant higher ratio of cTfh1/cTfh17 cells (**Figure 2G**) that, in the disaggregated analysis, was particularly characteristic of the STAT1 GOF and the CVID_dys_ patients but was not detected in the CVID_no-dys_, CTLA4, CD25def, and STAT5b patients (**Figures 2G, H**). Overall, these results demonstrate that the dysregulation of the immune system in these patients encompasses naïve, activated, central memory, Treg, cTfh, and their subsets of CD4^+^ T cells and that the cTfh1/cTfh17 ratio discriminates between CVID patients with and without dysregulation.

### CD8^+^ T Cells From PIRD patients Exhibit Increased Frequencies of Activated, TEM, and TEMRA Cells

**Figure 3:**
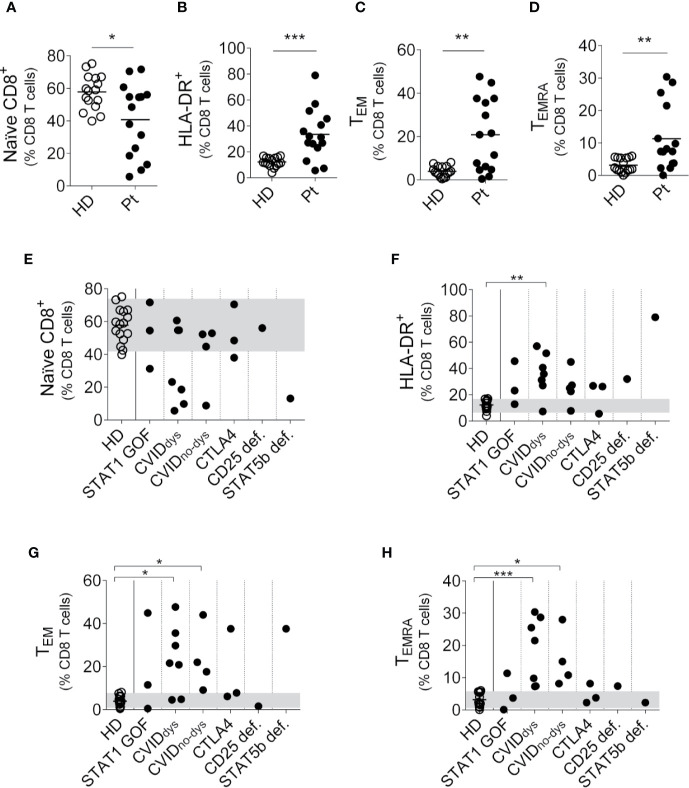
Relative frequencies of CD8^+^ T cells subsets in PIRD patients. The frequency of CD45RA^+^CD27^+^CD8^+^ (naïve) T cells **(A)**, HLA-DR^+^CD8^+^ (activated) T cells **(B)**, CD45RA^-^CD27^-^CD8^+^ (T_EM_) cells **(C)** and CD45RA^+^CD27^-^CD8^+^ (T_EMRA_) cells **(D)** in a cohort of 16 HD and 15 patients with dysregulatory syndrome PID were depicted. Horizontal lines represent the mean of each group. The distribution of the frequencies of CD45RA^+^CD27^+^CD8^+^ (naïve) T cells **(E)**, HLA-DR^+^ CD8^+^ (activated) T cells **(F)**, CD45RA^-^CD27^-^CD8^+^ (T_EM_) cells **(G)** and CD45RA^+^CD27^-^CD8^+^ (T_EMRA_) cells **(H)** in each group of this cohort was also depicted. P1 (STAT1 GOF) and P11 (CVID) were under treatment (as detailed in **Supplementary Table 1**). Gray areas indicate the 10^th^ and 90^th^ percentiles of HD for each parameter. An unpaired parametric *t*-Student test with Welch´s correction was used in **(A–C)**. An unpaired non-parametric *t*-Student test with Mann-Whitney U test was used in **(D)**. a non-parametric test with Dunn’s *post hoc* was used in **(E–H)**. *p < 0.05; **p < 0.01; ***, p < 0.001.

Then, we performed an analysis of the CD8^+^ T cell subsets using the gating strategy outlined in **Supplementary Figure 1A** and observed that, analyzed as a group and similarly to what we observed in CD4^+^ T cells, PIRD patients exhibited a reduced frequency of naïve CD8^+^ T cells (**Figure 3A**) and an increased frequency of activated (HLA-DR^+^) CD8^+^ T cells (**Figure 3B**). In addition, contrary to what we observed in the CD4^+^ T cell compartment, these patients exhibited significantly increased frequencies of CD8^+^ T_EM_ cells (**Figure 3C**) and CD8^+^ T_EMRA_ cells (**Figure 3D**) with no differences in the frequency of T_CM_ cells (*not shown*). A disaggregated analysis of the patients according to their mutation revealed that regardless of their mutation, most patients exhibited this skew toward an activated phenotype in the CD8 T cell compartment characterized by more activated and effector memory CD8^+^ T cells (**Figures 3E–H**). Therefore, our results demonstrate that the dysregulation of the immune system in these patients encompasses naïve, activated, effector memory and terminal effector subsets of CD8^+^ T cells.

### B Cells Subsets Varies Among Patients With Immune Dysregulation

**Figure 4:**
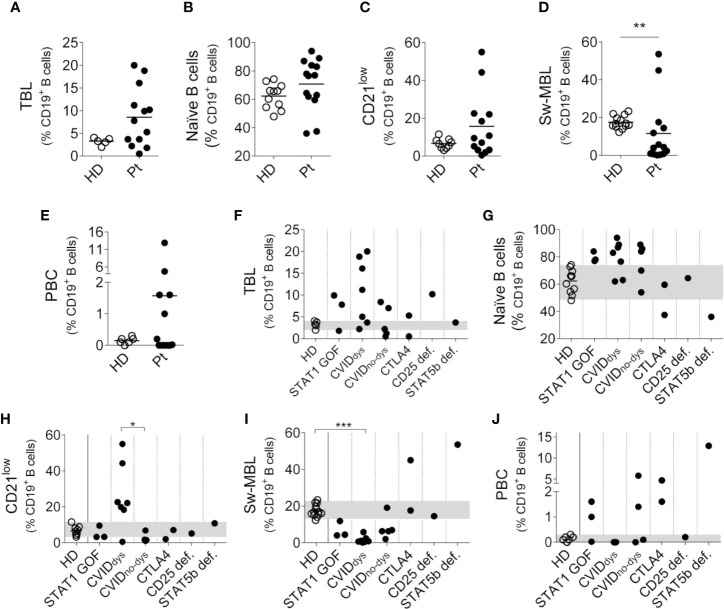
Relative frequencies of B cells subsets in PIRD patients. The frequency of CD38^++^CD24^++^ cells (TBL, **A**), IgD^+^CD27^-^ cells (naïve B cells, **B**) CD19^+^CD21^low^CD38^+/-^ cells (CD21^low^ B cells, **C**), IgD^-^IgM^-^CD38^+/-^ cells (Sw-MBL, **D**) and CD38^++^CD27^++^ cells (PBC, **E**) in a cohort of HD (*n* = 5 for TBL, *n* = 11 for naïve B cells, *n* = 9 for CD21^low^ B cells, *n* = 13 for Sw-MBL and *n* = 6 for PBC) and 14 patients with dysregulatory syndrome PID were depicted. Horizontal lines represent the mean of each group. The distribution of the frequencies of CD38^++^CD24^++^ cells (TBL, **F**), IgD^+^CD27^-^ cells (naïve B cells, **G**) CD19^+^CD21^low^CD38^+/-^ cells (CD21^low^ B cells, **H)**, IgD^-^IgM^-^CD38^+/-^ cells (Sw-MBL, **I**), and CD38^++^CD27^++^ cells (PBC, **J**) in each group of this cohort were also depicted. P1 (STAT1 GOF) and P11 (CVID) were under treatment (as detailed in **Supplementary Table 1**). Gray areas indicate the 10^th^ and 90^th^ percentiles of HD for each parameter. An unpaired non-parametric *t*-Student test (Mann-Whitney U test) was used in **(A–E)**; a non-parametric test with Dunn’s *post hoc* test was used in **(F–J)**. *p < 0.05; **p < 0.01; ***p < 0.001.

We also analyzed the B cell compartment in the patients, but we were unable to perform this analysis in P7 because she was under Rituximab treatment and then she underwent hematopoietic stem cell transplantation. To identify the different B cell subpopulations, we use the gating strategy outlined in **Supplementary Figure 1C**. Analyzed as a group, most PIRD patients exhibited an increased frequency of TBL (**Figure 4A**), with no major changes in the frequency of naïve B cells (**Figure 4B**) and CD21^low^ B cells (with the exception of five CVID patients, **Figure 4C**), a significant reduced frequency of Sw-MBL (**Figure 4D**) and variable frequencies of PBC (**Figure 4E**). The disaggregated analysis of the patients according to their mutation revealed that, compared to HD, two STAT1 GOF, five CVID, one CTLA4 and the CD25 deficient patients presented increased frequencies of TBL (**Figure 4F**). Also, the STAT1 GOF and five of the CVID patients presented increased frequencies of naïve B cells in blood, while one CTLA4 and the STAT5b patients presented reduced frequencies of naïve B cells in blood (**Figure 4G**). The disaggregated analysis of the patients revealed that the only group that exhibited higher frequencies of CD21^low^ B cells were the CVID_dys_ patients, and this increase was significant when compared to CVID_no-dys_ patients (**Figure 4H**). In addition, the CVID_dys_ patients presented a significant reduction in the frequency of Sw-MBL cells, while one of the CTLA4 and the STAT5b deficient patients exhibited increased frequencies of these cells in blood (**Figure 4I**). Moreover, no differences between CVID patients with and without dysregulation were observed. In the PBC compartment, we observed a strikingly high frequency of PBC in the STAT5b deficient patients and a less marked increase in two of three STAT1 GOF, the two CTLA4, and two of the four CVID_no-dys_ patients (**Figure 4J**). Overall, our results unravel a profound dysregulation in the B cell compartment, in some cases characterized by a prevalence of most immature/naïve B cells (such as in STAT1 GOF and CVID patients with dysregulation) and in other cases characterized by a prevalence of most activated/differentiated B cells (such as in STAT5b deficient and one CTLA4 patient, and in some CVID patients without dysregulation).

### Correlation Between cTfh17 Cells and Sw-MBL Cells

**Figure 5:**
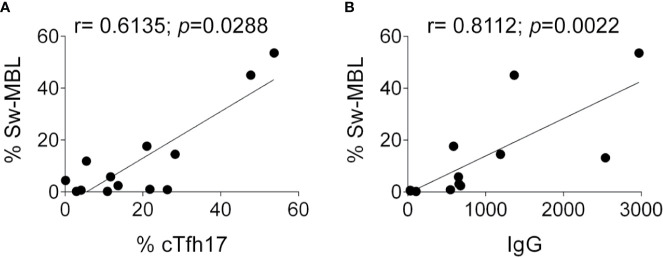
Correlation between cTfh17 cells, Sw-MBL cells, PBC, and serum IgG in PIRD patients. Correlation graphs between the frequency of cTfh17 cells and Sw-MBL **(A)**, and between Sw-MBL and IgG in serum **(B)** were depicted. Statistical analysis was performed using Spearman’s rank correlation and the values of r and *p* are indicated in each graph.

As Tfh cells are specialized in providing help to B cells during the GC reaction and considering the altered frequencies in the cTfh and B cell compartments detected in these patients, we evaluated whether the frequency of cTfh is associated with the frequency of different B cell subsets. Although there was no correlation between cTfh and any B cell population (TBL, naïve B cells, CD21^low^ B cells, Sw-MBL, and PBC, *not shown*), we observed a significant positive correlation between the frequencies cTfh17 cells and Sw-MBL (**Figure 5A**) and between the frequency of Sw-MBL and serum IgG (**Figure 5B**). Overall, these results demonstrate that the dysregulation in the B cell compartment of the patients is associated with abnormalities in the cTfh cell subsets and that such alterations are associated with a disbalance in the amount of IgG in serum. Of note, in P9, P10, P13, and P15 we could evaluate their phenotypes prior and after treatment and observed no major differences in the T and B cells subsets.

## Discussion

PIRD constitute an evolving group of diseases that lead to an altered immune homeostasis resulting in inappropriate tolerance, autoimmunity, allergy and/or inflammation (1, 2, 12, 49, 50). In this work, we explored the T and B compartments of a cohort of 15 PIRD patients that share clinical features such as autoimmunity and recurrent infections. Although we used a limited number of patients within each group, our study highlights the utility of FC as a suitable tool to characterize different T and B cells subsets and their alterations in these diseases with clinically overlapping features. Also, our findings contribute to unravel phenotypic alterations that might be associated, at least in part, with some of the clinical manifestations observed in these patients.

Phenotype analysis evidenced that the main discriminating variable among these overlapping diseases were found within the CD4^+^ compartment. PIRD patients presented increased frequencies of cTfh cells, known to be effector T cells that provide B cell help which results in B cell expansion, immunoglobulin class switch, affinity maturation and secretion of high-affinity antibodies by PBC (32, 51, 52). Therefore, the abnormal frequencies of cTfh seen in patients likely impacts on B cell help for antibody production and contribute to their clinical picture, especially, in PIRD with autoimmune and inflammatory conditions (53, 54). Accordingly, increased frequencies of cTfh cells have been involved in the pathogenesis of several autoimmune diseases and their frequency positively correlated with serum autoantibody titers (35, 55). The B-cell lymphoma 6 protein (Bcl-6) transcription factor is essential for Tfh development (56, 57) and it has been observed that STAT5 protein is a negative regulator of Bcl-6 (58–61). Moreover, mouse models evidenced that STAT1 activity is essential for IL-6-mediated Bcl-6 induction for early Tfh differentiation (62). Therefore, our observations in PIRD patients might be the consequence of an altered signaling necessary to balance Tfh development, as STAT1 GOF and STAT5b patients may exhibit a constitutive Bcl-6 expression that shifts CD4 T cell differentiation to the Tfh lineage. In addition, cTfh17 and cTfh2 subsets are highly proficient cTfh effectors that promote immunoglobulin class switching and generation of Sw-MBL and PBC due to their high IL-21 secretion ability (28, 34, 35). We observed that some patients with PIRD displayed impaired frequency distribution of the cTfh1 and cTfh17 subsets with impaired cTfh1/cTfh17 ratio. In particular, STAT1 GOF and CVID patients with dysregulation exhibited low frequencies of cTfh17 but increased frequencies of cTfh1 cells, reduced frequencies of Sw-MBL and hypogammaglobulinemia (with the exception of P1 that coursed with autoimmune hepatitis and hypergammaglobulinemia). Conversely, the patient with the STAT5b mutation presented an opposite phenotype as the observed in the asymptomatic mutation carrier of the CTLA-4 family (P8), who presented high frequencies of Sw-MBL and never displayed hypogammaglobulinemia. Other two affected family members carrying the same CTLA-4 mutation (P6 y P7) presented with CVID phenotype. Strikingly, the values of cTfh1 and cTfh17 (and the corresponding ratio) within the CVID group without dysregulation were quite different from those of the CVID with dysregulation, for which their assessment may contribute to the differential diagnosis of both kind of CVID patients. Also, our results unraveled a positive correlation between cTfh17, Sw-MBL, and IgG in serum, suggesting that the assessment of the global frequency of cTfh cells does not contribute to the diagnosis. Instead, the assessment of the cTfh1 and cTfh17 distribution (and eventually, the calculation of the cTfh1/cTfh17 ratio) in patients with PIRD may contribute to a better classification of these heterogeneous group of PID, as increased proportions of cTfh2 and cTfh17 may contribute to the exacerbated humoral responses and autoantibody production as seen in other autoimmune diseases (34–36).

Other interesting findings were that all PIRD patients presented low frequencies of naïve CD4^+^ and CD8^+^ cells with a concomitant increased frequency of activated CD4^+^ and CD8^+^ T cells. Within the subsets of memory T cells, the patients presented increased frequencies of T_CM_ CD4^+^ T cells and T_EM_ and T_EMRA_ CD8^+^ T cells, which was not present in the CVID group without dysregulation. Since memory T cells display a lower activation threshold, it is possible that the increased frequencies of memory T cells in the PIRD patients also contribute to the autoimmune picture observed in these patients (63, 64). Therefore, our results indicate that PIRD patients exhibit a skew toward an activated/memory phenotype within the T cell compartment, which probable is the consequence of the underlying mutation and the chronic antigenic stimulation that they experience during recurrent infections, and that might be associated with their inflammatory symptoms and autoimmune conditions.

The IL-2/CD25/STAT5b signaling axis plays a non-redundant role driving Treg cells development (65, 66) and activates B lymphocyte-induced maturation protein-1 (Blimp-1), thus inhibiting Bcl-6 and regulating B cell responses (61, 66–70). Interestingly, most patients of our cohort exhibited low frequencies of Treg (they were absent in the STAT5b and CD25 patients). Moreover, CTLA-4 is expressed in Treg cells and plays a key role in their suppressive function (16, 71, 72) These findings suggest that a disbalance in these cells might also be involved in the generation of auto-antibodies in PIRD patients that escape from the tolerance checkpoints of the GC. Furthermore, the low frequencies of Treg may likewise affect “Regulatory Follicular T cells” (Tfr) that specifically regulate Tfh activity, B cell activation and GC reactions (55, 73–75), further increasing the susceptibility to the development of autoimmunity in PIRD patients, as an imbalance between Tfh and Tfr has been associated with autoimmune diseases (76, 77).

Regarding CD21^low^ B cells, these cells have been found in increased proportions in patients with several autoimmune diseases, such as SLE and Sjögren’s syndrome (78). It has been proposed that they develop from MBL that have undergone chronic stimulation associated with cTfh1 and IFN-γ dysregulation (79–81). However, in our study, we observed increased frequencies of CD21^low^ B cells only in CVID_dys_ patients that also exhibited increased frequencies of TBL and reduced frequencies of Sw-MBL as described by others (82–85). Therefore, the concomitant accumulation of CD21^low^ B cells and immature/naïve cells in detriment of activated/effector cells that we observed in these patients, together with additional observations that indicate that CD21^low^ B cells are IgM^+^IgD^+^CD27^-^ support the idea that CD21^low^ B cells exhibit phenotypic features more closely related to naïve B cells or B cells in early stages of activation.

Our results demonstrate that patients with PIRD course with dissimilar phenotypes in the T and B cell compartments. Therefore, immunophenotyping of peripheral blood cells may provide physicians the opportunity to differentiate them and to timely administer targeted therapies to alleviate their conditions (86). Accordingly, administration of CTLA-4-Ig therapy promotes a decrease in cTfh and the skew toward cTfh1 improving patients’ medical condition, a fact that indicates that cTfh monitoring by FC constitutes a useful and sensitive manner to assess response to treatment (87). Also, Sirolimus (SRL), received by P5, P7, P11 and P15 during follow up, blocks mTOR and partially restores Treg function through a FOXP3-independent mechanism (88) and inhibits the differentiation of naive T cells into functional cTfh cells, antagonizing Th1 and Th17 responses (88, 89). Nonetheless, besides clinical improvement after receiving SRL, these patients did not exhibit a restoration in their frequency of Treg nor a normalization of the frequencies of cTfh and cTfh1/cTfh17 ratio. These patients have received SRL since its indication and treatment has been sustained since then, for which we were unable to perform new analysis in treatment-free samples. However, these results confirmed the same laboratory phenotype alterations seen prior to treatment. Also, anti-CD20 mAb (received by P7, P9 and P10) has been successfully used to ameliorate non-infectious complications in CVID_dys_ patients not only due to B cells depletion but also promoting a rise in the frequency of Treg cells and a normalization of the Th1/Th2 ratio in memory CD4^+^ T cells (90). However, the only effect we could confirm during the follow up of these patients was the B cell depletion in peripheral blood. Finally, corticosteroids affect immature and mature T cells by repressing maturation, differentiation, proliferation and inducing apoptosis, promoting a shift to Th2 cells through negative regulation of T-bet (91, 92). Patients that received these drugs (P1, P5, P12, and P13) did not evidence such phenotypical changes. Accordingly, our data of the analysis of PIRD patients’ phenotypes along time, reinforces the idea that the observed alterations would be consequence of their underlying disease/mutation and the chronic activation/dysregulation state, and not secondary to the treatment administered.

In summary, PIRD patients exhibit a skew toward an activated/memory phenotype within the CD4^+^ and CD8^+^ T cell compartment, accompanied by abnormal frequencies of Tregs, cTfh and their cTfh1 and cTfh17 subsets that likely impact on B cell help for antibody production and may contribute to their autoimmune and inflammatory conditions. Therefore, assessment of cTfh1 and cTfh17 cells by FC constitutes a simple and straightforward tool for these complex clinical entities that may impact early diagnosis and patients’ treatment.

## Data Availability Statement

The raw data supporting the conclusions of this article will be made available by the authors, without undue reservation.

## Ethics Statement

The studies involving human participants were reviewed and approved by Comité de ética e investigación del Hospital de Niños “Ricardo Gutierrez”. Written informed consent to participate in this study was provided by the participants’ legal guardian/next of kin. Written informed consent was obtained from the individual(s), and minor(s)’ legal guardian/next of kin, for the publication of any potentially identifiable images or data included in this article.

## Author Contributions

MC performed and designed most of the experiments, analyzed the data, and wrote the manuscript. MM contributed experimentally. LB contributed with critical discussions and handling of the patients. NZ and MG conceived, designed, and supervised the study. All the authors reviewed the manuscript. All authors contributed to the article and approved the submitted version.

## Funding

This work was supported by a grant from the National Agency for Promotion of Science and Technology from Argentina (ANPCYT) to NZ and the LASID fellowship with the support of Shire to MM.

## Conflict of Interest

The authors declare that the research was conducted in the absence of any commercial or financial relationships that could be construed as a potential conflict of interest.

## Abbreviations

Bcl-6, B-cell lymphoma-6; Blimp-1, B lymphocyte-induced maturation protein-1; BSA, bovine serum albumin; cTfh, circulating follicular helper T cells; CTLA-4, cytotoxic T lymphocyte–associated antigen-4; CVID, common variable immune deficiency; FC, flow cytometry; GC, germinal center; GOF, gain of function; HD, healthy donors; mAb, monoclonal antibodies; MBL, memory B cells; mTOR, mammalian-target-of-rapamycin; PBC, plasmablast cells; PBMC, peripheral blood mononuclear cells; PBS, phosphate-buffered saline solution; PID, primary immunodeficiencies; PIRD, primary immune regulatory disorders; SRL, Sirolimus; Sw-MBL, switched memory B cells; TBL, transitional B lymphocytes; Tfh, follicular helper T cells; Treg, regulatory T cells.
