# Supplementary material for: Immune Monitoring of Patients With Primary Immune Regulation Disorders Unravels Higher Frequencies of Follicular T Cells With Different Profiles That Associate With Alterations in B Cell Subsets
Source: Front Immunol. 2020 Oct 29;11:576724. doi: 10.3389/fimmu.2020.576724 (PMC7658009; doi:10.3389/fimmu.2020.576724)

**Supplementary Figure 1.A)** Gating strategy used to identify T cellsubsets. **B)** Gating strategy used to identify Treg. **C**)Gating strategy used to identify B cell subsets.

**A**


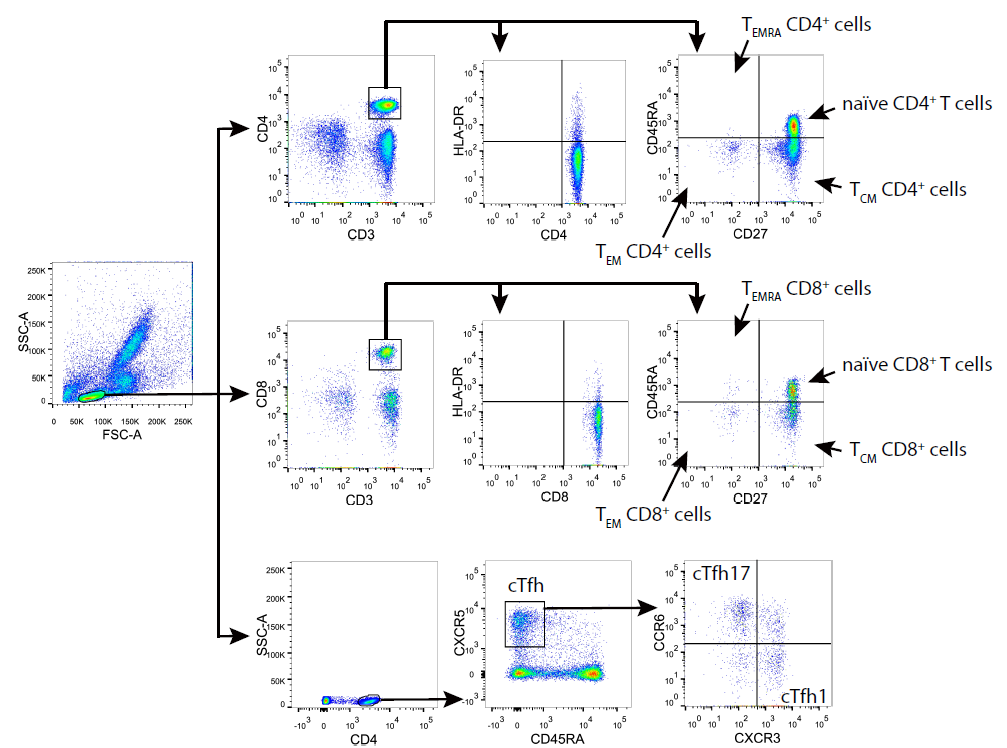


**B**


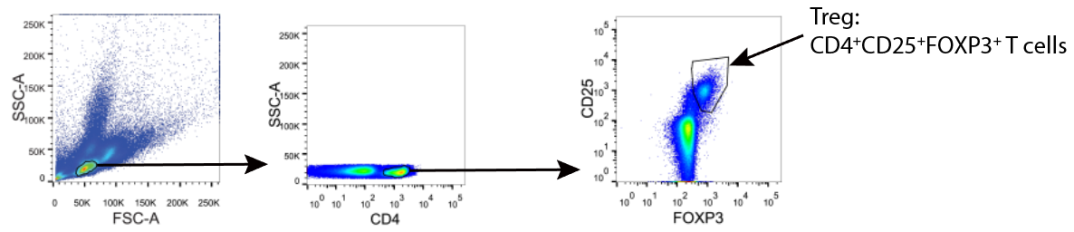


**C**


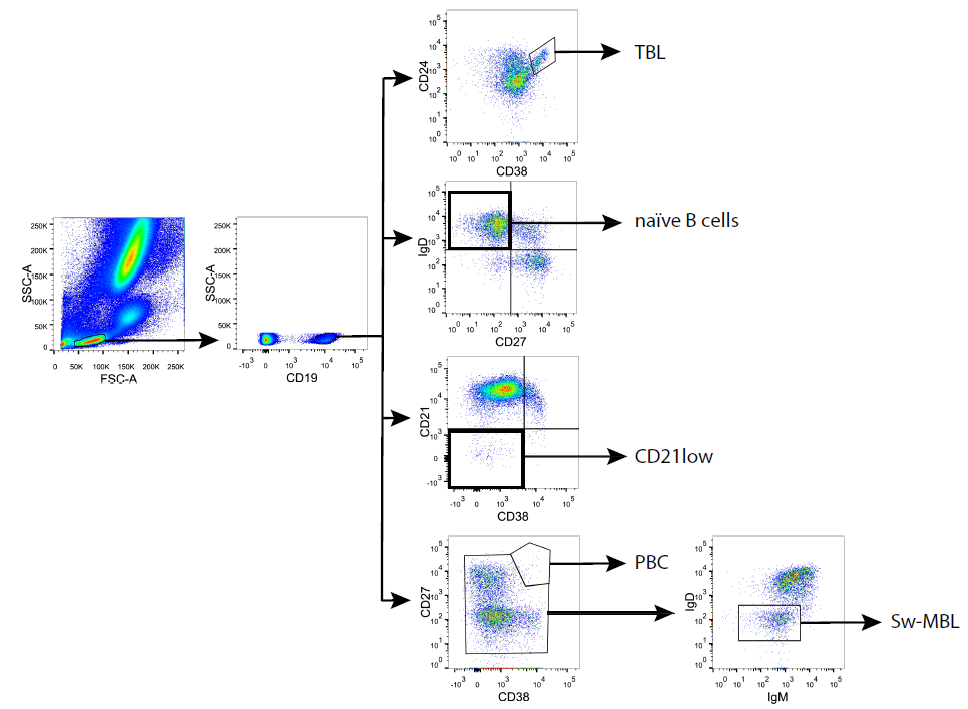

Supplement: Supplementary file 1 [file DataSheet_1.docx]
