# Supplementary material for: Immune Monitoring of Patients With Primary Immune Regulation Disorders Unravels Higher Frequencies of Follicular T Cells With Different Profiles That Associate With Alterations in B Cell Subsets
Source: Front Immunol. 2020 Oct 29;11:576724. doi: 10.3389/fimmu.2020.576724 (PMC7658009; doi:10.3389/fimmu.2020.576724)
Supplement: Supplementary file 2 [file Table_1.docx]

**Table 1: *Clinical features of patients included in this study***

|  | PT  (AGE) | Organ AUTOIMMUNITY | cytopenia | ENTEROPATHY | INFECTIONS | LYMPHOPROLIFERATIVE DISEASE | OTHER | treatment  (IMS/IMM) | MOLECULAR DIAGNOSIS (MUTATION) |
| --- | --- | --- | --- | --- | --- | --- | --- | --- | --- |
| STAT 1 GOF | P1**^a^** (21) | Autoimmune hepatitis, desquamative erythema, alopecia | No | No | CMC, *Staphilococcus* *sp*. bacteriemia, disseminate ringworm, *Trichomonas vaginalis*, *Microsporidium canis* | No | No | Corticosteroids, Azathioprine  * | Heterozygous (p.R274Q) |
|  | P2 (14) | No | Mild lymphopenia | No | BCGosis, bronchiolitis, pneumonia meningitis (*M. tuberculosis*), CMC, bacteriemia  (*S. viridans*) | No | Loss of maturational patterns | No | Heterozygous (p.Q167H) |
|  | P3 (33) | Hypothyroidism, erythema, asthma, athopy | Mild lymphopenia | No | Pulmonar histoplasmosis, recurrent pneumonia, CMC, Herpes labialis, thrush, *Mycobacterium* sp. | No | No | No | Heterozygous (p.F172L) |
| STAT5b def. | P4 (27) | Hypothyroidism, eczema, psoriasis, celiac disease, alopecia | Lymphopenia | Chronic diarrhea | Severe varicella with cutaneous infection | No | Severe growth failure | No | Homozygous (p.F646S) |
| CD25 def. | P5 (12) | Eczema, alopecia | Mild lymphopenia | Chronic diarrhea | Varicella, *Herpes Zoster,* pneumonia (CMV) | Follicular bronchiolitis | No | Sirolimus  Corticosteroids  Ig replacement  * | Homozygous (p.Y41S) |
| CTLA4 haplo | P6 (19) | No | Thrombocytopenia, lymphopenia | Chronic diarrhea | Recurrent respiratory infections | Intestinal follicular,  lymphoid hyperplasia | No | Azathioprine  Ig replacement  * | Heterozygous (p.L141P) |
|  | P7 (15) | No | Thrombocytopenia, anemia | No | Oral thrush | No | No | Rituximab, Sirolimus,  Ig replacement  ** | Heterozygous (p.L141P) |
|  | P8 (46) | No | No | Diarrhea/  constipation | Recurrent respiratory infections | Intestinal follicular  lymphoid hyperplasia | No | No | Heterozygous (p.L141P) |
| CVID_dys_ | P9 (46) | No | Lymphopenia | Chronic diarrhea | Pneumonia, sinusitis, suppurative otitis, tuberculosis | Adenomegaly | Abdominal granulomas | Rituximab  Corticosteroid (Sporadic)  Ig replacement  * | N/D |
|  | P10  (46) | No | Lymphopenia | Chronic diarrhea | Pneumonia  *Campilobacter spp*. infection,  *Clostridium difficile* infection | No | No | Rituximab  Ig replacement  * |  |
|  | P11 (48) | Hypothyroidism,  DBT2, vitiligo | Mild thrombocitopenia | Ulcerative colitis-like, chronic diarrhea | Meningitis with no germ rescue, measles, recurrent pneumonia, *Candida* esophagitis | Mild splenomegaly | BQT | Mesalazine, Hidroxicloroquine  Sirolimus  Ig replacement  ** |  |
|  | P12 (14) | No | Thrombocytopenia | No | Otitis, pneumonia, sinusitis | GLILD, Splenomegaly | No | Corticosteroid  Ig replacement  * |  |
|  | P13 (17) | No | Thrombocytopenia, neutropenia, anemia | No | Pneumonia, suppurative otitis, *Parvovirus* and *Picornavirus* infection | Splenomegaly | No | Corticosteroid  Micofenolate  Ig replacement  * |  |
|  | P14 (18) | No | Thrombocytopenia | No | Pneumonia, sinusitis, suppurative otitis | Splenomegaly | Arthritis with no germ rescue | Ig replacement |  |
|  | P15 (17) | No | Thrombocytopenia, neutropenia | No | LIP | Splenomegaly | Lung/  Hepatic granulomas | Corticosteroid, Sirolimus  Ig replacement  ** |  |
| CVID _no-dys_ | P16  (13) | No | No | No | Pneumonia, recurrent obstructive  bronchitis | No | No | Ig replacement | N/D |
|  | P17  (28) | No | No | No | Sinusitis, suppurative otitis | No | No | Ig replacement |  |
|  | P18  (77) | No | No | No | Pneumonia, bronchitis, sinusitis, ocular toxoplasmosis | No | No | Ig replacement |  |
|  | P19  (18) | No | No | No | Pneumonia, otitis | No | No | Ig replacement |  |
|  | P20  (16) | No | No | No | Pneumonia, suppurative otitis | No | No | Ig replacement |  |

**^a^** deceased; **IMS/IMM,** immunosupresive/immunomodulator treatment. **BQT**, bronchiectasis; **CMC**, mucocutaneous candidiasis; **DBT2**, Type 2 diabetes mellitus; **GLILD**, Granulomatous-Lymphocytic Interstitial Lung Disease; **LIP**, Lymphoid Interstitial Pneumonia; **Ig,** Immunoglobulin (intravenous or subcutaneous). **N/D,** *not determined*

* Evaluated prior and after a minimum of one year inmunosupressive/inmunomodulator treatment suspension**.**

**Evaluated prior and during Sirolimus treatment. P7 was excluded of the LB analysis due to Rituximab and received HSCT.
